# The citrus flavonoid naringenin confers protection in a murine endotoxaemia model through AMPK-ATF3-dependent negative regulation of the TLR4 signalling pathway

**DOI:** 10.1038/srep39735

**Published:** 2016-12-22

**Authors:** Xin Liu, Ning Wang, Shijun Fan, Xinchuan Zheng, Yongjun Yang, Yuanfeng Zhu, Yongling Lu, Qian Chen, Hong Zhou, Jiang Zheng

**Affiliations:** 1 Medical Research Center, Southwest Hospital, the Third Military Medical University, Chongqing, 400038, China; 2 Department of Pharmacology, College of Pharmacy, the Third Military Medical University, Chongqing 400038, China

## Abstract

Excessive activation of the TLR4 signalling pathway is critical for inflammation-associated disorders, while negative regulators play key roles in restraining TLR4 from over-activation. Naringenin is a citrus flavonoid with remarkable anti-inflammatory activity, but the mechanisms underlying its inhibition of LPS/TLR4 signalling are less clear. This study investigated the molecular targets and therapeutic effects of naringenin *in vitro* and *in vivo*. In LPS-stimulated murine macrophages, naringenin suppressed the expression of TNF-α, IL-6, TLR4, inducible NO synthase (iNOS), cyclo-oxygenase-2 (COX2) and NADPH oxidase-2 (NOX2). Naringenin also inhibited NF-κB and mitogen-activated protein kinase (MAPK) activation. However, it did not affect the IRF3 signalling pathway or interferon production, which upregulate activating transcription factor 3 (ATF3), an inducible negative regulator of TLR4 signalling. Naringenin was demonstrated to directly increase ATF3 expression. Inhibition of AMPK and its upstream calcium-dependent signalling reduced ATF3 expression and dampened the anti-inflammatory activity of naringenin. In murine endotoxaemia models, naringenin ameliorated pro-inflammatory reactions and improved survival. Furthermore, it induced AMPK activation in lung tissues, which was required for ATF3 upregulation and the enhanced anti-inflammatory activity. Overall, this study reveals a novel mechanism of naringenin through AMPK-ATF3-dependent negative regulation of the LPS/TLR4 signalling pathway, which thereby confers protection against murine endotoxaemia.

Toll-like receptors 4 (TLR4) are type I transmembrane receptors primarily expressed by innate immune cells^1^,^2^. The engagement of TLR4 activation by microbe-derived pathogenic molecules, such as lipopolysaccharide (LPS), leads to activation of multiple intracellular signalling pathways and transcription events, which further induce essential antimicrobial activities^3^. However, excessive TLR4 activation may trigger the pathogenesis of sepsis, autoimmune diseases and other inflammatory-associated disorders^4^,^5^. Therefore, a variety of negative feedback regulatory mechanisms have evolved to restrain TLR4 from over-activation^6^. Activating transcription factor 3 (ATF3), a member of the ATF/CREB family, functions as a key inducible negative regulator of TLR4 signalling^6^,^7^. ATF3 primarily binds to the CREB-ATF elements within the promoter region and dampens the transcription of inflammatory genes by histone deacetylase recruitment and chromatin remodelling^7^. Furthermore, ATF3 was recently found to negatively regulate NF-κB in RAW 264.7 cells via direct interaction with the p65 subunit^8^.

The anti-inflammatory effect of ATF3 is well documented. ATF3-deficient macrophages produce remarkably lower levels of proinflammatory cytokines such as IL-12p40, TNF-α and IL-6 upon LPS stimulation^7^. ATF3-null mice are also more susceptible to endotoxic shock challenge^9^. More recently, ATF3 was demonstrated to be activated by high-density lipoprotein and to mediate the anti-inflammatory reprogramming of macrophages, which suggests that ATF3 activation may be a promising therapeutic target in inflammatory diseases^10^. ATF3 is commonly regarded as a feedback regulator and induced by TLR-dependent activation by bacterial-derived stimuli in macrophages. For example, the production of interferon (IFN) is essential for the full expression of ATF3^11^. However, the regulation pattern of ATF3, in addition to LPS stimulation, has not been well elucidated.

Natural products derived from food, fruits and traditional herbs have been traditionally used to treat infection and inflammatory diseases^12^. Naringenin and its glucoside naringin are flavonoid-type natural compounds that can be purified from grapefruit and other citrus species. Emerging evidence has revealed that naringenin displays anti-inflammatory and antioxidant activities, which are thought to be required for its efficacy in treating inflammatory-associated atherosclerosis, arthritis and metabolic syndrome^13^,^14^. However, the molecular mechanisms underlying the anti-inflammatory properties of naringenin require further elucidation. In the present study, we evaluated the anti-inflammatory properties and life-protective efficacy of naringenin in LPS-stimulated macrophages and in a murine endotoxaemia model. We also investigated the underlying mechanism of naringenin-associated inflammation control, which may provide more evidence for verifying potent therapeutic targets of anti-inflammatory flavonoids.

## Results

### Naringenin inhibits the production of proinflammatory mediators in LPS-stimulated murine macrophages

**Figure 1:**
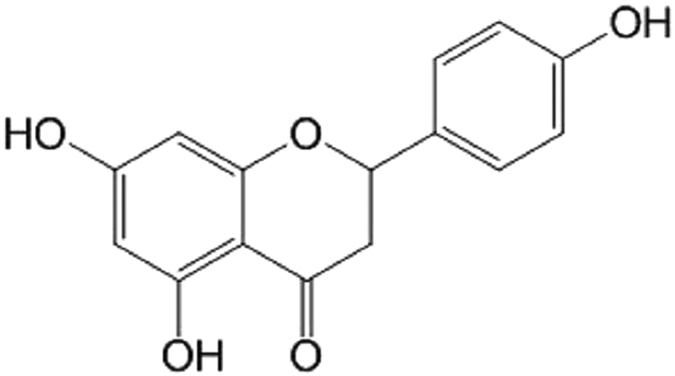
Chemical structure of naringenin.

**Figure 2:**
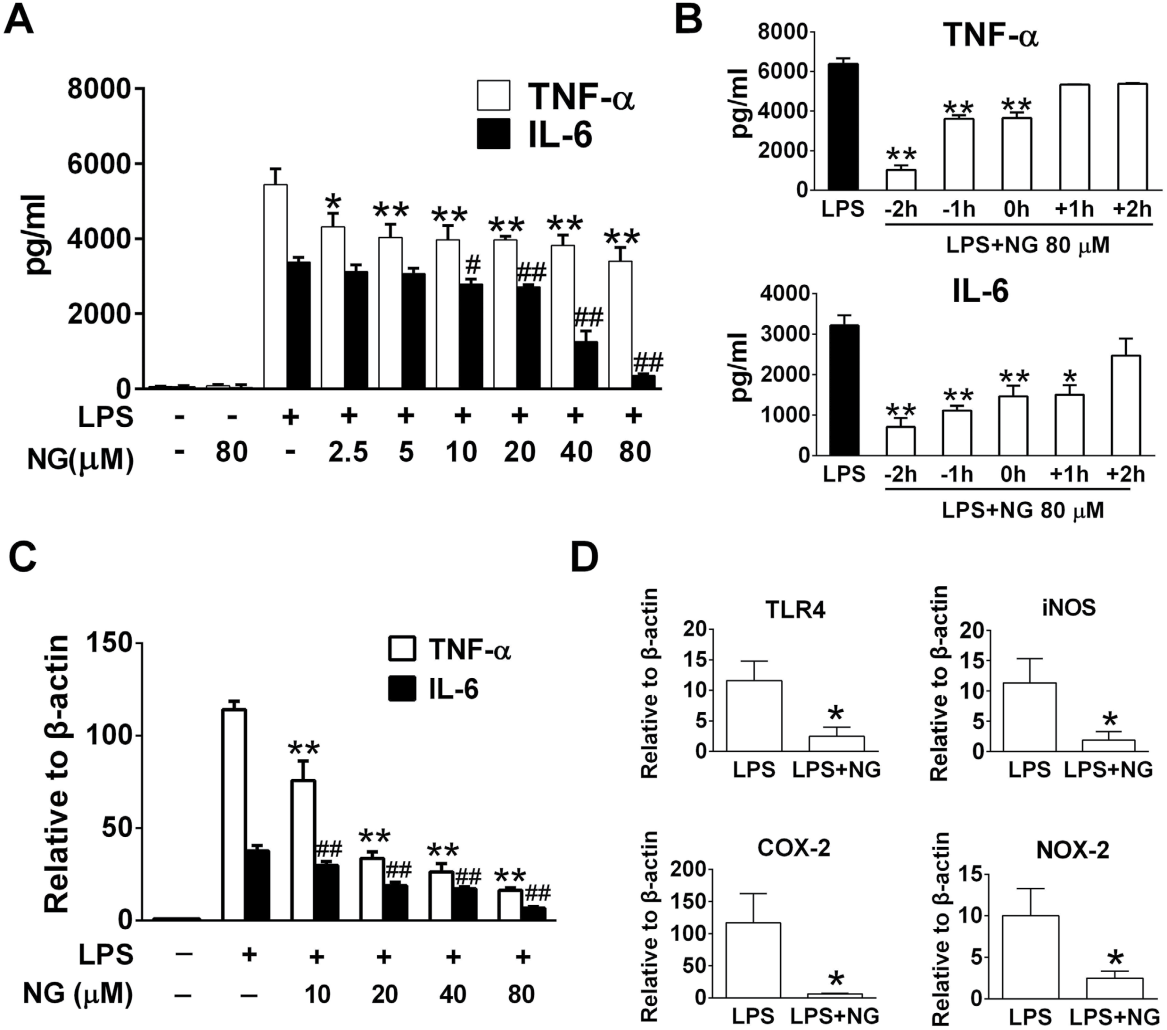
Naringenin inhibits the upregulation of proinflammatory mediators induced by LPS in RAW 264.7 cells. (**A**) TNF-α and IL-6 production affected by naringenin in series concentrations. Cells were treated with 80 μM NG, LPS or LPS with NG (2.5, 5, 10, 20, 40 and 80 μM) for 12 h. Supernatant TNF-α and IL-6 levels were detected by ELISA (n = 3). *P < 0.05, **P < 0.01 *vs* LPS (TNF-α), ^#^*P* < 0.05; ^##^*P* < 0.01 *vs* LPS (IL-6). (**B**) Time-dependent analysis of TNF-α and IL-6 production upon naringenin treatment. Cells were treated for 12 h with LPS or with NG, which was added 2 and 1 h before LPS or 0, 1 or 2 h post-LPS stimulation. Supernatant TNF-α and IL-6 levels were detected by ELISA (n = 3). *P < 0.05, **P < 0.01 *vs* LPS. (**C**) TNF-α and IL-6 mRNA expression affected by series concentrations of naringenin. Cells were treated with LPS or with NG (10–80 μM) for 4 h. TNF-α and IL-6 mRNA was detected by real-time PCR (n = 3). **P < 0.01 *vs* LPS (TNF-α), ^##^*P* < 0.01 *vs* LPS (IL-6). (**D**) Inhibition of mRNA expression of TLR4, iNOS, COX-2 and NOX-2 by naringenin. Cells were treated with LPS alone or with NG for 4 h. mRNA expression of TLR4, iNOS, COX-2 and NOX2 was detected by real-time PCR (n = 3). *P < 0.05 *vs* LPS. Naringenin is abbreviated as NG. The concentrations of LPS and NG were 100 ng/ml and 80 μM unless indicated.

To evaluate the anti-inflammatory properties of naringenin, a typical pure flavanone compound (Figs 1 and S1), we detected proinflammatory cytokines in LPS-stimulated murine macrophages. Naringenin was shown to suppress TNF-α and IL-6 release in a dose-dependent manner in RAW 264.7 cells (Fig. 2A). The inhibitory effect was not due to cytotoxicity, as naringenin did not affect cellular viability below 160 μM (Figure S2). Similar inhibitory effects were observed in primary murine macrophages (Figure S3). The anti-inflammatory effects of naringenin were also time-dependent, and pre-incubation demonstrated increased efficacy (Fig. 2B). Moreover, naringenin significantly suppressed the mRNA expression of TNF-α and IL-6 in LPS-stimulated RAW 264.7 cells or BMDMs (Figs 2C and S4). It also markedly attenuated TLR4, inducible NO synthase (iNOS), cyclo-oxygenase-2 (COX2) and NADPH oxidase-2 (NOX2) expression levels that were upregulated by LPS (Fig. 2D). These results indicated that naringenin effectively suppressed the proinflammatory response in LPS-treated murine macrophages.

### Naringenin suppresses activation of the NF-κB and MAPK signalling pathways in LPS-treated RAW 264.7 cells

**Figure 3:**
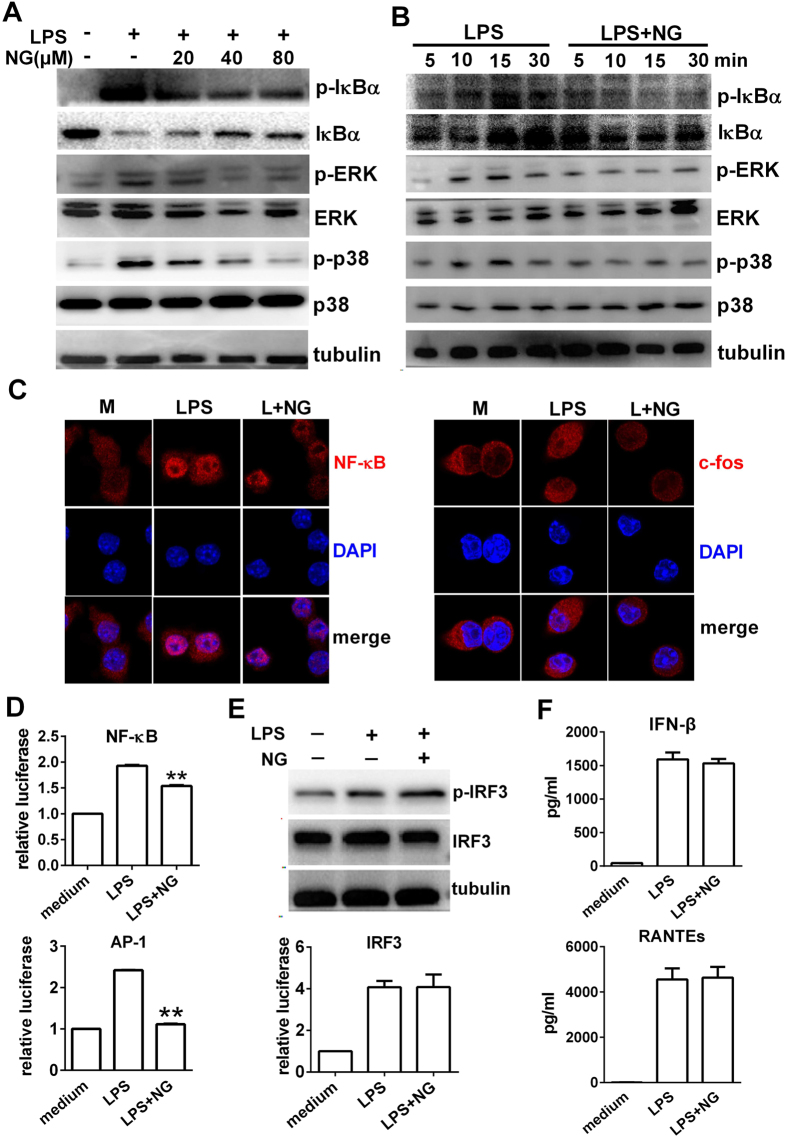
Naringenin inhibits MAPK and NF-κB pathways in LPS-treated RAW 264.7 cells. (**A**,**B**) Time- and dose- dependent inhibition on MAPK and NF-κB activation by naringenin. Cells were treated with LPS alone or with 20, 40 and 80 μM NG for 30 min (**A**). Cells were treated with LPS alone or with NG for 0, 5, 15 and 30 min (**B**). Protein levels of pIκBα, IκBα, pERK, ERK, p-p38, p38 and tubulin were detected by western blot. (**C)** Inhibition of nuclear translocation of NF-Kb p65 and c-fos by naringenin. Cells were treated with LPS alone or with NG for 2 h. Nuclear translocation of NF-κB p65 and c-fos were detected by immunofluorescence. (**D**) Inhibition of reporter activity of NF-kB and AP-1 by naringenin. Cells transfected with NF-κB and AP-1 reporter plasmids and treated with LPS alone or together with NG for 6 h. Relative reporter activity was detected by the luciferase assay (n = 3). **P < 0.01 *vs* LPS. (**E**) Activity of IRF3 detection. Cells were treated with LPS alone or together with NG; IRF3 phosphorylation was detected by western blot (upper), and the relative reporter activity of IRF3 was detected by a luciferase assay (lower). (**F**) Production of supernatant IFN-β and RANTES levels. Cells were treated as in (**E**) for 12 h and supernatant IFN-β and RANTES levels were detected by ELISA (n = 3). Naringenin is abbreviated as NG. The concentrations of LPS and NG were 100 ng/ml and 80 μM unless indicated.

We next investigated whether naringenin affected activation of the MAPK-, NF-κB- and IRF3-dependent pathways, which contribute to the production of proinflammatory cytokines. Herein, naringenin inhibited the phosphorylation of IκB-α, p38 and ERK in both dose- and time-dependent manners (Fig. 3A,B). Similar inhibitory effects were observed in BMDMs (Figure S5). Moreover, naringenin markedly suppressed the nuclear translocation of NF-κB and c-fos (a subunit of the AP-1complex and downstream of p38 and ERK) in LPS-treated RAW 264.7 cells (Fig. 3C). Naringenin also significantly inhibited the elevated transcriptional activity of NF-κB and AP-1, as shown by a luciferase reporter activity assay (Fig. 3D). In contrast, naringenin did not reduce the phosphorylation of IRF3 or decrease its transcriptional activity in LPS-stimulated macrophages (Fig. 3E). Moreover, naringenin was unable to inhibit the expression of IFN-β or regulated on activation, normal T cell expressed and secreted (RANTES), two proinflammatory mediators upregulated by activation of IRF3 (Fig. 3F). Therefore, our results suggest a selective inhibition of TLR4-dependent signalling by naringenin in LPS-stimulated macrophages.

### Naringenin upregulates ATF3 expression to mediate the inhibition of TLR4 dependent signalling and pro-inflammatory reactions

**Figure 4:**
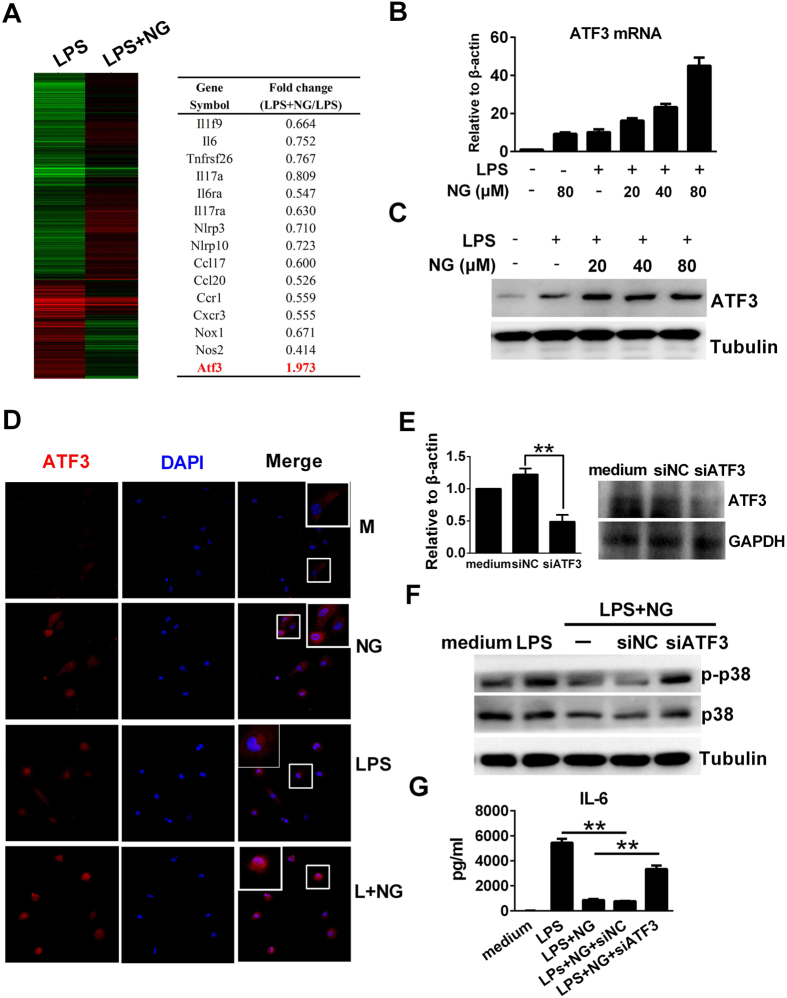
Naringenin upregulates ATF3 expression in RAW 264.7 cells, which is required for its anti-inflammatory action. (**A**) ATF3 expression as demonstrated in the transcriptome assay. Cells were treated with LPS or LPS plus NG for 4 h. The gene expression ratio of LPS + NG/LPS was analysed by a transcriptome assay. (**B)** Naringenin induces ATF3 mRNA expression. Cells were treated with NG, LPS and LPS plus NG (20, 40 and 80 μM) for 4 h. ATF3 mRNA was detected by real-time PCR (n = 3). (**C**) Naringenin induces ATF3 protein expression. Cells were treated with LPS alone or with NG (20, 40 and 80 μM) for 4 h. The protein level of ATF3 was detected by western blot. (**D**) Naringenin enhances cellular staining of ATF3. Cells were treated with NG, LPS or LPS plus NG for 4 h. Intracellular ATF3 was detected by immunofluorescence. Uncropped images are presented in Supplementary Figure S8A. (**E**) ATF3 knockdown by siRNA. Cells were transfected with control siRNA (siNC) or ATF3 siRNA (siATF3) for 24 h. Then, ATF3 mRNA was detected by PCR (n = 3, **P < 0.01). The protein level of ATF3 was detected by western blot. (**F**,**G**) ATF3 knockdown dampens the anti-inflammatory activity of naringenin. Wild-type cells and siNC- or siATF3-pretreated cells were treated with LPS alone or LPS plus NG. Plasma protein was collected 30 min after LPS treatment, and p-p38, p38 and tubulin were detected by western blot (**F**). Uncropped images are presented in Supplementary Figure S8A. Supernatants were collected 12 h after LPS treatment, and the IL-6 level was detected by ELISA (**G**). n = 3, **P < 0.01. Naringenin is abbreviated as NG. The concentrations of LPS and NG were 100 ng/ml and 80 μM unless indicated.

To explore the underlying inhibitory mechanisms against LPS, RNA-Seq was used to detect transcriptome profiling in RAW 264.7 cells. In our study, naringenin co-treatment resulted in a significant downregulation of proinflammatory mediators compared to LPS stimulation alone. In contrast, the expression of ATF3, a key negative regulator of the TLR4 signalling pathway, was upregulated by naringenin in murine macrophages (Figs 4A and S6A). We further determined that both the mRNA and protein levels of ATF3 were upregulated by naringenin alone or upon co-treatment with LPS in RAW 264.7 cells (Fig. 4B–D). Furthermore, ATF3 siRNA was transfected to downregulate its expression (Fig. 4E). In ATF3-knockdown macrophages, naringenin demonstrated a lower ability to inhibit the phosphorylation of p38 and the production of IL-6 compared with wild-type macrophages or macrophages transfected with scrambled siRNA (Fig. 4F,G). These results indicate that ATF3 may be involved in mediating the anti-inflammatory reactions of naringenin in LPS-treated macrophages.

### Naringenin upregulates ATF3-dependent anti-inflammatory activity by inducing calcium-dependent AMPKα activation

**Figure 5:**
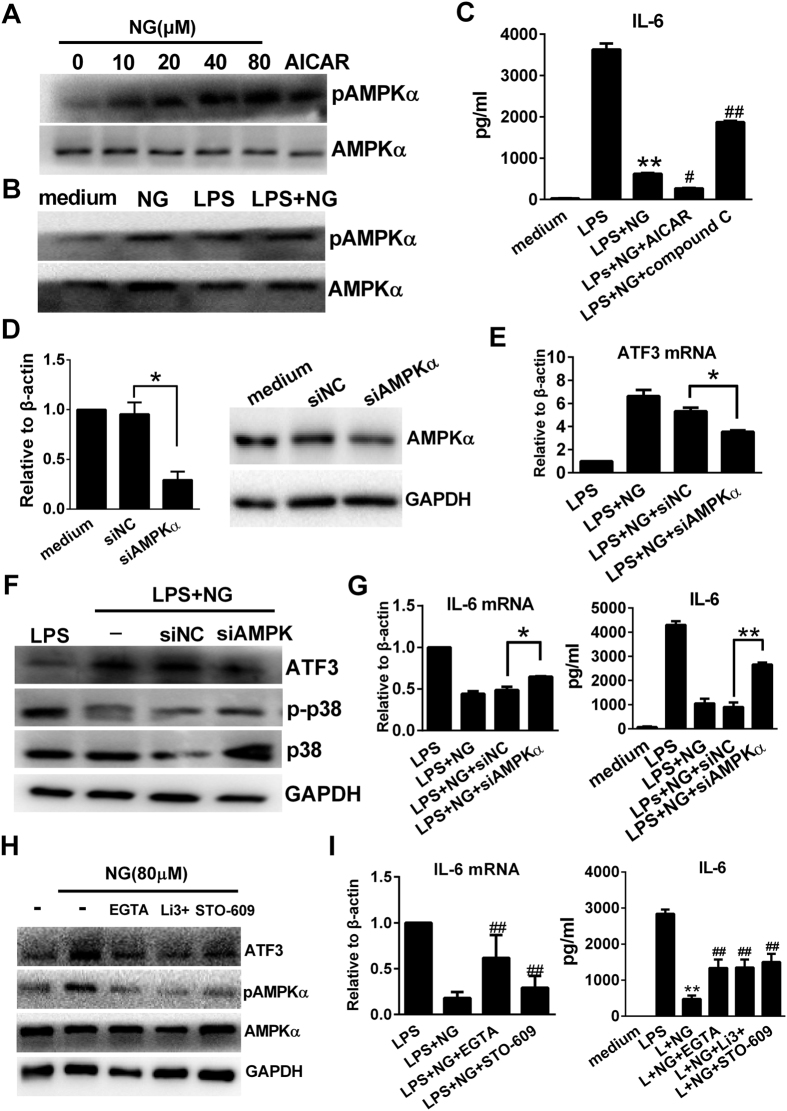
Naringenin activates AMPKα to mediate ATF3 upregulation in RAW 264.7 cells. (**A**) Dose-dependent induction of AMPK phosphorylation by naringenin. Cells were treated with NG (0, 10, 20, 40 and 80 μM) or 1 mM AICAR for 1 h. The phosphorylation of AMPKα was detected by western blot. (**B**) Naringenin enhances AMPK phosphorylation with LPS. Cells were treated with NG, LPS or LPS plus NG for 1 h. AMPKα phosphorylation was detected by western blot. (**C**) Modulation of AMPK affects IL-6 suppression by naringenin. Cells were treated with NG alone or in combination with 1 mM AICAR or 2 μM compound C before stimulation with LPS. Supernatant IL-6 was detected by ELISA (n = 3). **P < 0.01 *vs* LPS; ^#^*P* < 0.05, ^##^*P* < 0.01 *vs* LPS + NG (**D**) AMPK knockdown by siRNA. Cells were transfected with control siRNA (siNC) or AMPKα siRNA (siAMPKα) for 24 h. Then, AMPKα mRNA was detected by PCR (n = 3, *P < 0.05). The protein level of ATF3 was detected by western blot. Uncropped images are presented in Supplementary Figure S8B. (**E**–**G**) ATF3 expression and anti-inflammatory activity affected by AMPKα siRNA. Wild-type cells and siNC- or siAMPKα-transfected cells were treated with LPS alone or LPS plus NG. The mRNA expression of ATF3 was detected by RT-PCR (**E**) (n = 3, *P < 0.05). Then, ATF3, p-p38, p38 and tubulin were detected by western blot (**F**). The mRNA and protein levels of IL-6 were detected by RT-PCR and ELISA (n = 3, *P < 0.05, **P < 0.01). (**H–I**) Effects of calcium and CaMKKβ inhibition on cytokine production and ATF3/AMPKα activation. Cells were pretreated with 5 mM EGTA, 10 μM LiCl3 (Li3+) or 1 μM STO-609 before treating with NG alone or in combination with LPS. mRNA expression of IL-6 was detected by RT-PCR. The supernatant level of IL-6 was detected by ELISA n=3, **P < 0.01 vs LPS, ^##^*P* < 0.01 vs LPS + NG. ATF3, p-AMPK, AMPK and GAPDH were detected by western blot. Uncropped images are presented in Supplementary Figure S8B. Naringenin is abbreviated as NG. The concentrations of LPS and NG were 100 ng/ml and 80 μM unless indicated.

We next investigated the possible upstream regulators that lead to ATF3 upregulation. In our study, we observed that naringenin could independently enhance AMPKα phosphorylation in murine macrophages, similar to the AMPKα agonist AICAR (Fig. 5A). Moreover, the combined use of LPS and naringenin further increased AMPKα phosphorylation in murine macrophages (Figs 5B and S6B). Then, we found that co-treatment of AICAR with naringenin further decreased IL-6 production, while an AMPK inhibitor compound C partly restored IL-6 release when co-treated with naringenin (Fig. 5C). We next downregulated AMPKα expression by siRNA transfection in RAW 264.7 cells (Fig. 5D) and observed that the naringenin-induced ATF3 upregulation was significantly inhibited in AMPKα-knockdown macrophages (Fig. 5E,F). Moreover, naringenin-mediated suppression of p38 phosphorylation and IL-6 production were also attenuated by AMPKα knockdown (Fig. 5F,G). These data indicated that AMPKα activation was induced by naringenin and required for ATF3 upregulation and the subsequent anti-inflammatory activity. Calcium influx and CaMKKβ activation act as major upstream regulators of AMPK activation. Herein, we also demonstrated that inhibition of extracellular calcium influx (by a calcium chelator or a calcium channel inhibitor) or suppression of CaMKKβ activation (STO-609) reduced AMPK activation, downregulated ATF3 expression and resulted in marked increase in IL-6 production (Fig. 5H,I). The above data indicate that naringenin may trigger calcium influx and activate CaMKKβ and AMPK to mediate the ATF3-dependent anti-inflammatory activity.

### Naringenin improves survival and suppresses inflammatory responses in a murine endotoxaemia model

**Figure 6:**
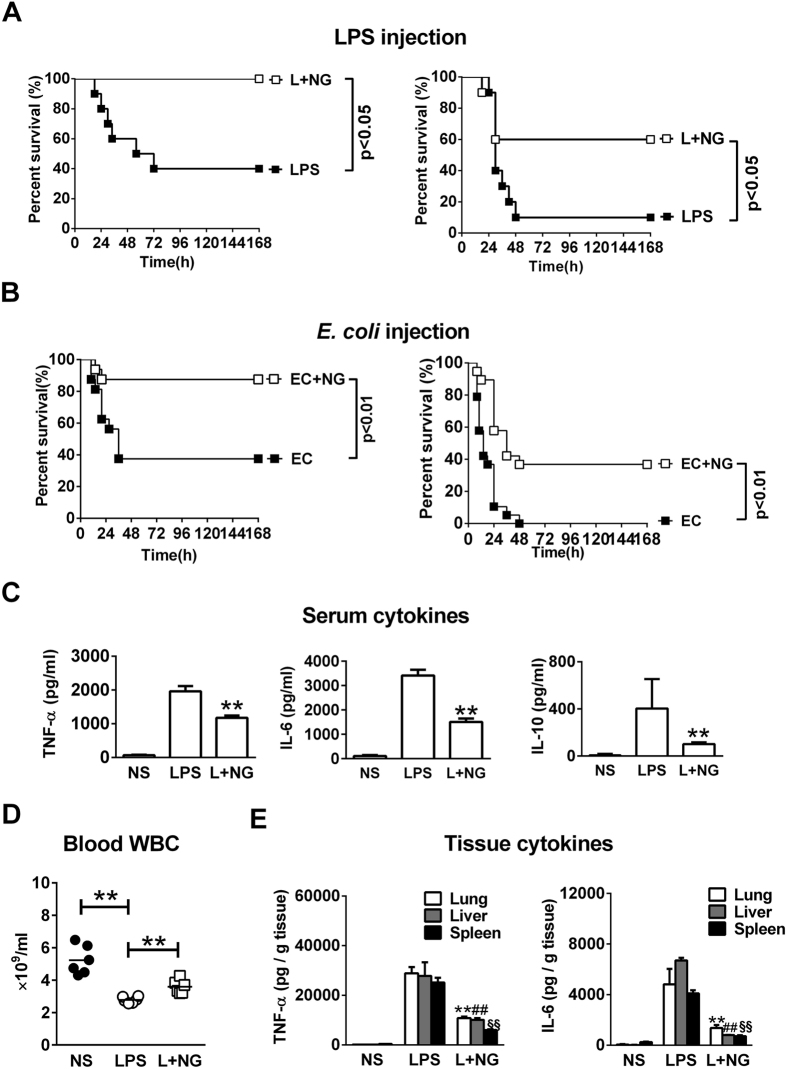
Naringenin improves survival and ameliorates systemic and tissue inflammatory reactions in endotoxaemia mice. (**A**) Survival analysis of the LPS-injected mice. Mice were intraperitoneally injected with 10 mg/kg (left) or 20 (right) mg/kg LPS (■) or in combination with NG (L + NG, □). Survival was observed for 7 days (n = 10). (**B**) Survival analysis in the bacteria injection model. Mice were intraperitoneally injected with 1.0 × 10^10^ CFU/kg (left) or 2.0 × 10^10^CFU/kg (right) heat-killed *E. coli* (■) or in combination with NG (L + NG, □). Survival was observed for 7 days (n = 16). (**C**–**E**) Detection of cytokines and cell counts in blood or tissue samples. Mice were injected with normal saline (NS), LPS or LPS with NG (L + NG). Samples from blood, lung, liver and spleen tissues were obtained 12 h after injection. Serum levels of TNF-α, IL-6 and IL-1β were detected by ELISA (**C**). Blood WBCs were detected using haematological analysers (**D**). **P < 0.01 *vs* LPS. The levels of TNF-α and IL-6 per gram protein (**E**) were detected in homogenates of the lung, liver and spleen (n = 3). **, ^##^ and ^§§^represent *P* < 0.01 *vs* LPS for lung, liver and spleen, respectively. Naringenin is abbreviated as NG. The doses of LPS and NG were both 10 mg/kg unless indicated.

To verify the anti-inflammatory activity of naringenin *in vivo*, the survival and proinflammatory responses in an LPS-challenged sepsis mice model was detected. In the survival analysis, injections of 10 or 20 mg/kg LPS resulted in the mortality rates of 60% or 90%, respectively, in the mice. Treatment with naringenin significantly reduced the mortality rates to 0% and 40%, respectively (Fig. 6A). Furthermore, heat-killed *E. coli* was used to mimic actual LPS challenge during infection. Intraperitoneal *E. coli* injection resulted in mild or severe mortality in mice, and naringenin also significantly increased the survival rates in both models (Fig. 6B). We next detected significant increases in serum TNF-α, IL-6 and IL-10 levels 6 h after LPS challenge, which was markedly reduced by naringenin treatment (Fig. 6C). In addition, LPS injection induced a rapid decrease in blood leukocytes in the whole blood, which was also reversed by naringenin administration (Fig. 6D). Finally, we detected elevations in TNF-α and IL-6 in homogenates of the lungs, liver and spleen. Treatment with naringenin markedly decreased the production of TNF-α and IL-6 in these organs (Fig. 6E).

### Naringenin upregulates AMPK-dependent ATF3 activation and mediates lung protection in the lung tissues of endotoxaemia mice

**Figure 7:**
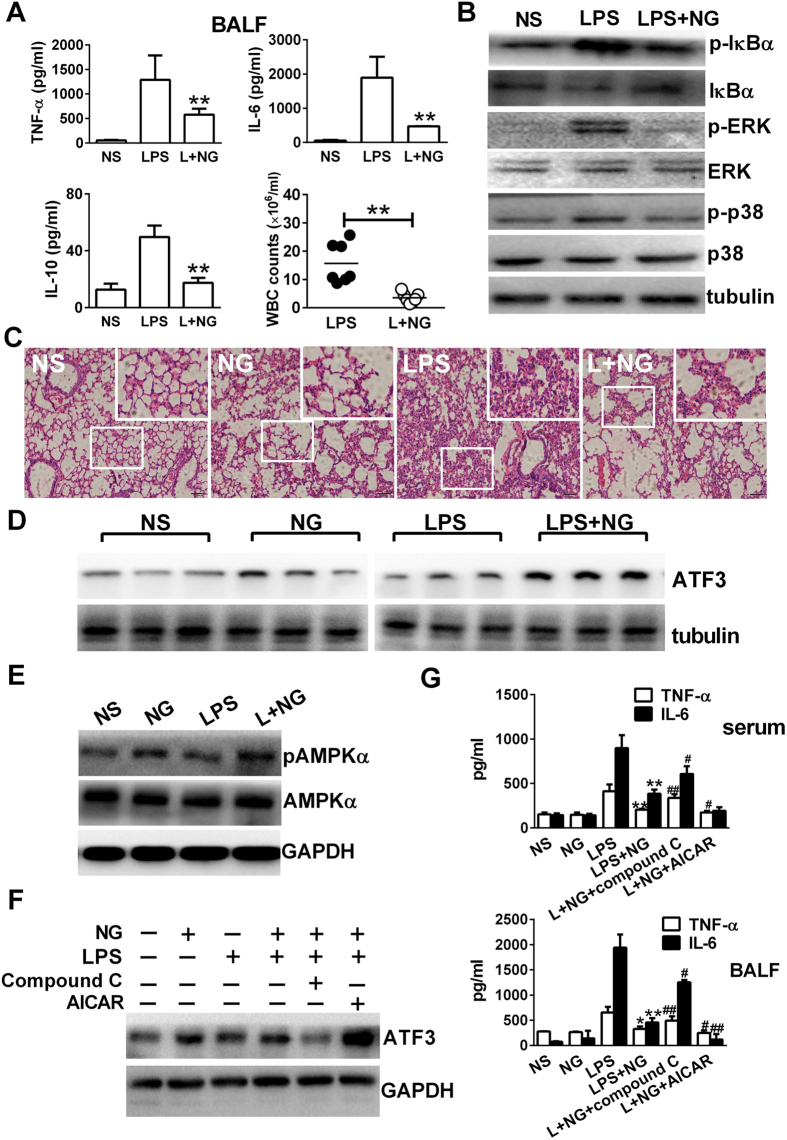
Naringenin upregulates ATF3 expression in lung tissues of LPS-challenged mice, which is AMPK dependent and required for limiting proinflammatory reactions. (**A**,**B**) Detection of cytokines and cell counts in BALF and Western blot assays for signalling molecules in murine lung tissues. Mice were injected with NS, LPS or LPS with NG. BALF or lung tissues were collected 12 h after LPS injection. TNF-α, IL-6 and IL-10 levels and WBC counts (n = 3) in the BALF were measured (**A**). Protein levels of p-IκBα, IκBα, ERK, pERK, p-p38, p38 and tubulin were detected by western blot (**B**). (**C**–**E**) Detection of histological changes, ATF3 expression and AMPK activation in murine lung tissues. Mice were injected with NS, NG, LPS or LPS with NG, and lung tissues were collected 12 h after injection. Histopathological changes (**C**) were observed, and the protein levels of ATF3 (**D**) or pAMPKα and AMPKα (**E**) were detected by western blot. Uncropped images are presented in Supplementary Figure S8C,D. (**F**,**G**) Effects of the co-injection of AMPK modulators on the levels of ATF3 and proinflammatory cytokines. Mice were injected with NS, NG, LPS, LPS plus NG, LPS plus NG and compound C (1 mg/kg) or LPS plus naringenin and AICAR (100 mg/kg). Blood, lung tissues and BALF were collected 12 h after injection. ATF3 expression in the lung tissues were detected by western blot (**F**). TNF-α and IL-6 levels in the serum or in the BALF (**G**) were detected by ELISA (n = 3). *P < 0.05, **P < 0.01 *vs* LPS; ^#^*P* < 0.05; ^##^*P* < 0.01 *vs* LPS+NG. Naringenin is abbreviated as NG. Broncho-alveolar lavage fluid is abbreviated as BALF. The doses of LPS and NG were both 10 mg/kg unless indicated.

The lungs are the most severely affected organs during sepsis and other acute inflammatory conditions. In this study, we observed elevations in TNF-α, IL-6 and IL-10 accompanied with increased leukocyte counts in broncho-alveolar lavage fluid (BALF) after LPS administration. However, serum levels of IFN-β and RANTEs were not affected (Figure S7). Consistently, naringenin inhibited increases in cytokines and leukocytes in the BALF (Fig. 7A). Then, we detected suppressed IκB-α, p38 and ERK activation in lung tissues by naringenin, indicating that the proinflammatory signalling induced by LPS was also suppressed by naringenin treatment (Fig. 7B). In histological analysis, LPS injection induced severe bleeding and pulmonary interstitial thickening in the lung tissue of mice. Treatment with naringenin resulted in markedly reduced tissue damage in the lungs (Fig. 7C). We next detected the activation of AMPK and ATF3 to determine their involvement in lung protection mediated by naringenin. In our study, naringenin alone or in combination with LPS increased ATF3 expression in the mouse lung tissues (Fig. 7D). Naringenin treatment also resulted in enhanced phosphorylation of AMPKα (Fig. 7E). Then, LPS-challenged mice were treated by naringenin with AICAR or compound C. The naringenin induced ATF3 upregulation in the lung tissue was enhanced by AICAR while attenuated by compound C (Fig. 7F). Accordingly, serum and BALF IL-6 levels were decreased by AICAR co-treatment but increased by compound C co-treatment (Fig. 7G). These results demonstrated that AMPK and ATF3 were activated by naringenin to mediate protection in the mice challenged with lethal LPS injection.

## Discussion

Excessive TLR4 activation in innate immune cells leads to uncontrolled proinflammatory responses, while intracellular negative regulators play a critical role in facilitating downregulation^1^,^6^. In the present study, we describe a new anti-inflammatory property of the citrus flavanone naringenin based on the upregulation of ATF3, a feedback negative regulator of the TLR4 dependent signalling pathway, in a calcium- and AMPK-dependent manner. Our results also demonstrate that naringenin administration could activate AMPK and upregulate ATF3 in the lung tissue of LPS-challenged mice, subsequently leading to the amelioration of lung injury and an improvement in survival. Therefore, the AMPK-ATF3 pathway may serve as an important drug target for flavonoid compounds to mediate anti-inflammatory activity.

Macrophages are the major source of various types of inflammatory mediators upon microbial stimuli, especially in systemic inflammatory disorders such as sepsis^15^. Our experiments demonstrated that naringenin exerted antagonistic effects on LPS-induced TNF-α and IL-6 expression in murine macrophages. Such results are in accordance with previous reports that showed that naringenin inhibits the production of proinflammatory cytokines and chemokines in LPS-stimulated epithelial cells and microglia cells^16^,^17^. Moreover, naringenin demonstrated inhibitory activity against enzymes (iNOS, COX-2 and NOX2), which are responsible for the production of secondary mediators in macrophages. It was previously reported that naringenin attenuated the hepatic expression of COX-2 and iNOS in carbon tetrachloride (CCL-4)-challenged rats^18^. However, naringenin was found unable to suppress the upregulation and activation of COX2 in LPS-stimulated microglia cells^17^. Such differences may be attributed to multiple mechanisms involved in the transcription of COX-2 expression in different cell types.

Cellular signalling pathways are activated downstream of TLR4, which enhance transcriptional events to upregulate the proinflammatory effectors. There are generally two signalling pathways activated by TLR4^2^,^19^. One is the MyD88-dependent pathway and involves NF-κB and MAPK activation. The other is the MyD88-independent pathway, which induces IRF3 activation and the production of IFNs, as well as IFN inducible mediators such as RANTES and IP-10^20^,^21^. Previously, naringenin was reported to inhibit the activation of MAPK and NF-κB signalling pathways in epithelia cells and dendritic cells, which was required to suppress inflammation or cell maturation^22^,^23^. However, it is not known whether naringenin affects the MyD88-independent pathway. In our study, we obtained similar results in murine macrophages that naringenin effectively attenuated the phosphorylation of IκB-α, ERK and p38. It also significantly reduced the nuclear distribution and transcriptional activity of NF-κB and AP-1. Interestingly, we found that naringenin did not affect the activation of IRF3 or inhibit the production of IFN-β and RANTES, which require IRF3 activation. To the best of our knowledge, this study indicates for the first time the lack of interference between naringenin and the IRF3 signalling pathway. Instead, naringenin is more capable of selectively inhibiting pro-inflammatory reactions involving mainly NF-κB and MAPK signalling.

To find the contributing factors underlying the inhibitory effects of naringenin, we performed a transcriptome analysis and screened out the enhanced expression of ATF3 upon naringenin co-treatment in LPS-stimulated macrophages. ATF3 is primarily induced by TLR stimulation as part of a negative-feedback loop of inflammation control^10^. Although the functional significance is controversial under different situations, increasing evidence suggests that ATF3 acts as a protective adaptive homeostatic mechanism that limits the inflammatory response by controlling the expression of a number of cytokines and chemokines in LPS-stimulated macrophages. For example, HDL mediates the reprogramming of macrophages and exerts broad anti-inflammatory actions through the induction of ATF3 expression^10^. In our study, we observed that naringenin enhanced the expression of ATF3 in both quiescent and LPS-activated macrophages. Moreover, genetic knockdown of ATF3 led to increased proinflammatory signalling transduction and cytokine production, which demonstrated the requirement of ATF3 upregulation in mediating the anti-inflammatory action of naringenin. It was previously reported that ATF3 is required for the inhibition of iNOS and COX2 activation in activated macrophages^24^,^25^. Therefore, the upregulation of ATF3 may also be necessary to explain the suppression of iNOS and COX2 mediated by naringenin.

AMPK is a crucial intracellular sensor under stress conditions. Recently, AMPK activation has appeared to be involved in cellular anti-inflammatory functions in macrophages^26^. However, AMPK is commonly linked with the NF-κB pathway to explain inflammation control^27^. Previously, naringenin was found to directly activate AMPK in vascular endothelial cells and muscle cells^28^,^29^. Therefore, we speculated that naringenin may induce ATF3 upregulation by activating AMPK. Our present work demonstrated for the first time that AMPK activation is essential for ATF3 induction and is also positively involved in mediating the anti-inflammatory action of naringenin in LPS-activated macrophages. Interestingly, we found that other flavonoids or polyphenols analogues of naringenin, such as quercetin, baicalin and resveratrol, could also induce AMPK phosphorylation and upregulate ATF3 expression (data not shown). In addition, quercetin and resveratrol, which are similar to naringenin, exert stronger activities to induce AMPK and ATF3 activation. Therefore, our findings may indicate a common feature of naringenin and other polyphenol analogues, which exert anti-inflammatory activities by activating the AMPK-ATF3 signalling pathway^30^,^31^.

In a recent study, p38 was demonstrated to be activated by naringenin and to mediate the upregulation of ATF3 in human colon cancer cells^32^. However, our results showed that naringenin inhibited p38 activation in LPS-treated macrophages, suggesting that p38 may not be required for ATF3 upregulation in macrophages. Instead, AMPK may be activated as a key upstream regulator for the upregulation of ATF3. More recently, it was reported that ATF3 induction by Toll-like receptors is strictly dependent on IFN-signalling^33^. In our study, naringenin failed to inhibit the activation of IRF3 or attenuate the production of IFN-β or RANTES. Such results also suggest that the MyD88-independent signalling pathway or IFN-β production may also mediate the upregulation of ATF3 induced by naringenin in macrophages. Phosphorylation of AMPK could be also induced by upstream kinases of Ca2+-calmodulin-dependent kinase kinase β (CaMKKβ) which phosphorylates and activates AMPK in response to increased intracellular calcium levels. Previously, naringenin was reported to increase the extracellular calcium influx in HEK293T cells^34^. In the present study, we found that extracellular calcium chelation or inhibition of CaMKKβ activation impaired the inhibition on IL-6 expression. Accordingly, the enhanced AMPK phosphorylation and ATF3 expression induced by naringenin were also suppressed. Other flavonoid compounds, such as saponarin, could also activate AMPK in a calcium-dependent manner^35^. Therefore, we speculate that naringenin may induce extracellular calcium influx and activate CaMKKβ, which thereby lead to AMPK activation and ATF3 upregulation.

LPS or heated-killed *E. coli* are strong inducers of endotoxaemia in animal models, which cause dramatic increases in TNF-α and IL-6 in the serum and major organs and thereby result in systemic inflammation and quick death in model animals. Moreover, the latter model was induced by injection of bacteria, which lacks viability. However, PAMPs such as LPS still exist, which are released or directly sensed to induce endotoxaemia or sepsis. Therefore, this model could better mimic the actual condition of endotoxaemia developed from bacterial infection. In our study, treatment with naringenin effectively suppressed the elevation of proinflammatory cytokines and improved the survival of mice injected with LPS and heated-killed *E. coli*. However, naringenin did not inhibit IFN-β and RANTES, which was consistent with cellular experiments and further indicate that the IRF3 pathway was also not affected *in vivo*. The lung is the most frequently affected organ after LPS injection and also closely related with increased mortality in mouse models^36^. Likewise, our experiments demonstrated that naringenin effectively attenuated cytokine levels and leukocyte infiltration in the lung tissue and in the BALF of LPS-challenged mice. Previously, it was found that transgenic overexpression of ATF3 specifically in macrophages resulted in marked attenuation of TNF-α and IL-6 expression in adipose tissue and peritoneal macrophages in response to saturated fatty acids/TLR4 signalling^37^. In our study, we also detected increased ATF3 expression and AMPK phosphorylation in the lung tissue of the model mice after naringenin treatment. Moreover, co-treatment with an AMPK activator enhanced the anti-inflammatory activity of naringenin, whereas an AMPK inhibitor functioned in an opposite manner. Overall, the *in vivo* data support our speculation that AMPK and ATF3 are required to mediate anti-inflammatory action by naringenin treatment.

**Figure 8:**
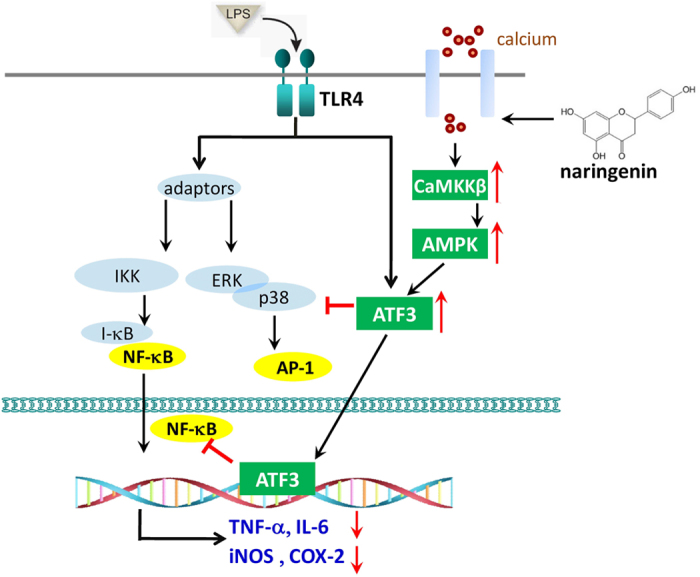
A schematic diagram describing the anti-inflammatory action of naringenin through AMPK-dependent upregulation of ATF3.

In summary, the findings of this study reveal a new function of naringenin that it suppresses inflammatory reactions in LPS-treated macrophages through AMPK-ATF3-dependent negative regulation on the TLR4 signalling pathway. Such effects were also required for naringenin to confer protection in the murine endotoxaemia model. A proposed schematic is presented as Fig. 8 to explain the mechanism, which offers additional avenues for studying the therapeutic potential of naringenin in treating inflammatory-associated disorders in the future.

## Materials and Methods

### Chemicals and Reagents

Naringenin was purchased from Source Leaf Biotech (Shanghai, China). Dulbecco’s modified Eagle’s medium (DMEM) and foetal bovine serum (FBS) were obtained from Gibco (Grand Island, NY, USA). *E. coli* lipopolysaccharide (LPS) O55:B5, ethylene glycol-bis (β-aminoethyl ether)-N,N,N’,N’-tetraacetic acid (EGTA), STO-609, LiCl3, AICAR and Compound C were purchased from Sigma (St. Louis, MO, USA). All other chemicals and solvents were of the best grade commercially available.

### Animals

Wide-type BALB/c mice (male, 6–8 weeks) were purchased from HFK Bioscience Co., Ltd. (Beijing, China) and housed under standard specific pathogen-free conditions with free access to food and water. All animal experiments were performed in accordance with the National and Institutional Guidelines for Animal Care and Use and approved by the Institutional Animal Ethics Committee of the Third Military Medical University.

### Endotoxaemia modelling and drug treatment

Murine endotoxaemia was modelled in BABL/c mice via a bolus intraperitoneal injection of NS or *E. coli* LPS O55:B5 or heat-killed *E. coli*. Then, a single dose of NS (containing 4% DMSO) or 10 mg/kg naringenin (containing 4% DMSO) was intraperitoneally injected alone or with 100 mg/kg AICAR or 1 mg/kg compound C. Survival was observed for up to 7 days, and samples from model mice for different tests were collected 12 h after modelling.

### Blood or tissue sampling

BALB/c mice were anesthetized under isoflurane inhalation (Keyuan Pharmaceutical Co., Ltd, Shandong, China). Blood samples were gathered via intracardiac puncture, and leukocytes were counted using a haemocytometer. The rest of the samples were centrifuged, and supernatants were stored at −70 °C for further cytokine assays. The entire right lung, liver and spleen organs were eviscerated 12 h after LPS injection and homogenized using 1 ml RIPA lysis solution (Beyotime, Jiangsu, China). The homogenates were centrifuged, and the supernatants were stored at −70 °C for further protein-based assays.

### Broncho-alveolar lavage fluid (BALF) sampling and cell counts

BALB/c mice were anesthetized under isoflurane inhalation. BALF samples were obtained as previously described^38^. Briefly, the trachea was exposed and cannulated with a small tube. The entire airway was lavaged by gently aspirating and pooled with 1 ml of sterile PBS. The BALF samples were collected, and leukocytes were counted in 50 μl aliquots using a haemocytometer. The remaining samples were centrifuged, and the supernatants were stored at −70 °C for cytokine assays.

### Cell culture and treatment

Murine peritoneal macrophages were obtained from peritoneal lavage in BALB/c male mice as previously described^39^. Briefly, BALB/c mice were euthanized, and 1 ml DMEM was injected into the intraperitoneal cavity. The abdomen was gently massaged for 1 min, the injected medium was aspirated, and the cell pellets were washed twice with DMEM containing 10% FBS. Then, peritoneal macrophages were plated in plates or dishes and cultured at 37 °C in a humidified incubator supplemented with 5% CO2. Murine bone marrow-derived macrophages (BMDMs) were isolated from the femurs of BALB/c mice and differentiated by supplementing with 50 ng/ml m-CSF for 3–5 days^40^. Primary macrophages or murine macrophage such as RAW 264.7 cells (ATCC, Manassas, VA, USA) were cultured in high-glucose DMEM supplemented with 10% FBS at 37 °C in a 5% CO2 humidified incubator. The cell viability was examined by trypan blue (Beyotime) staining, and the cell density was detected using a Bio-Rad cell counter and adjusted as indicated for further treatment. For controls of naringenin solvent, the LPS group contains DMSO up to 0.08% while the medium group remained untreated.

### Cell viability assay

The cytotoxicity of macrophages following treatment with naringenin was determined using an MTT assay. Cells were diluted to 1 × 10^5^ /ml and seeded into 96-well culture plates. Then, naringenin (0, 20, 40, 80, 160, 320 and 640 μM) was added and incubated for 24 h. MTT solution was added to the cultured cells and incubated at 37 °C for 4 h. Then, 150 μl DMSO was added, and the absorption values were detected at 550 nm wavelength using a Multi-mode plate reader (Thermo, USA).

### Western blot analysis

For cell-based analysis, macrophages (1 × 10^6^ /ml) were cultured in T25 flasks and collected after 30 min or as indicated after LPS treatment. Then, cells were lysed with plasma and a nuclear protein extraction kit (Beyotime) containing a protease cocktail and phosphatase inhibitors (Roche, Basel, Switzerland). For tissue-based analysis, the total protein was prepared as mentioned in Section 2.4. Then, cellular or tissue proteins were separated by SDS-PAGE and transferred onto PVDF membranes (Millipore, Billerica, MA, USA). Blots of plasma protein were incubated with primary antibodies (1:1000 dilutions) for p38, p-p38, ERK, pERK, IRF3, pIRF3, ATF3, pAMPKα, AMPKα and tubulin (Cell Signalling, Danvers, MA, USA) at 4 °C overnight. Then, the blots were further incubated with HRP-conjugated secondary IgG antibodies (1:2000 dilutions, Cell Signalling) at 37 °C for 1 h. Chemiluminescence images were developed with a SuperSignal Sensitivity Substrate kit (Pierce, Rockford, IL, US) and detected via a ChemiDoc XRS imaging system (Bio-Rad, Hercules, CA, USA).

### Real-time PCR assay

Macrophages (1 × 10^6^ /ml) were cultured in 6-well plates and treated as indicated, and then collected 4 h after LPS treatment. Total RNA was extracted using a TRIzol reagent (Roche) and reverse transcribed into cDNA with a ReverTra Ace-α-RNA easy kit (TOYOBO, Japan). The cDNA templates were mixed with SYBR Green PCR Mastermix (TOYOBO) and primers for TNF-α, IL-6, TLR4, iNOS, COX2, NOX2, IFN-β, RANTES, ATF3, and β-actin (Sequences listed in Table S1). Quantitative real-time PCR was performed using an iCycler Thermal Cycler (Bio-Rad).

### siRNA Transient Transfection

ATF3 and AMPKα were transiently knocked down by siRNA transfection according to the manufacturer’s instructions. Briefly, RAW264.7 cells were cultured to 70% confluence. Negative control (NC) siRNA or siRNA for ATF3 and AMPKα (Santa Cruz Biotechnology, USA) were mixed with Lipofectamine 3000 transfection reagent (Invitrogen, USA) and added to the medium. After 24 h of transfection, the culture medium was replaced, and further treatment was performed.

### Luciferase Reporter Gene Assay

RAW 264.7 cells (1 × 10^5^/ml) were cultured in 24-well plates and transfected with plasmid pGM-IRF3-Lu (Yesen Biotech, Shanghai, China) pGL6-NFκB-lu, pGL6-AP-1-Lu (Beyotime) using an X-treme GENE HP DNA Transfection Reagent (Roche) for 24 h. Then, LPS was added for 6 h of incubation treatment. The luciferase activity was analysed using a firefly luciferase assay kit (Beyotime) and detected using a luminometer. Relative luciferase light units were normalized to untreated cells.

### Immunofluorescence Assay

Macrophages (1 × 10^4^ /ml) plated in glass-bottom culture dishes were treated as indicated and collected 1 h after LPS treatment. Cells were fixed with 4% paraformaldehyde (Boster, China) for 10 min and permeabilized with Triton X-100 (Sigma) for 5 min, followed by incubation with NF-κBp65, c-fos and ATF3 primary antibodies (Cell Signalling) at 4 °C overnight. Then, the blots were further incubated with a Cy3-conjugated secondary IgG antibody (Cell Signalling), and the nuclei were stained with DAPI (Beyotime). Fluorescence images were captured using an LSM 780 confocal microscope (Zeiss, Germany).

### ELISA assays

Supernatants from cultured macrophages were directly collected 12 h after LPS treatment. Serum and BALF supernatant samples of sepsis mice were prepared as previously indicated. The levels of TNF-α, IL-6, IL-10, IFN-β and RANTES were detected with ELISA kits (eBioscience, San Diego, CA, USA) as indicated by the manufacturer’s instructions.

### Statistical analysis

Quantitative data are expressed as the mean ± standard deviation (SD). Student’s *t*-tests was used for comparisons between two groups. One-way ANOVA followed by the Bonferroni post hoc correction were used for multiple comparisons. Differences with *P* values less than 0.05 and 0.01 were considered statistically significant.

## Additional Information

**How to cite this article:** Liu, X. *et al*. The citrus flavonoid naringenin confers protection in a murine endotoxaemia model through AMPK-ATF3 dependent negative regulation of the TLR4 signalling pathway. *Sci. Rep.* **6**, 39735; doi: 10.1038/srep39735 (2016).

**Publisher's note:** Springer Nature remains neutral with regard to jurisdictional claims in published maps and institutional affiliations.

## Supplementary Material

Supplementary Figures and Tables
