# Supplementary material for: The citrus flavonoid naringenin confers protection in a murine endotoxaemia model through AMPK-ATF3-dependent negative regulation of the TLR4 signalling pathway
Source: Sci Rep. 2016 Dec 22;6:39735. doi: 10.1038/srep39735 (PMC5177915; doi:10.1038/srep39735)
Supplement: Supplementary Figures and Tables [file srep39735-s1.doc]

**Supplementary Materials**

**The citrus flavonoid naringenin confers protection in a murine endotoxaemia model through AMPK-ATF3-dependent negative regulation of the TLR4 signalling pathway**

Xin Liu^a^, Ning Wang^a^, Shijun Fan^a^, Xinchuan Zheng^a^, Yongjun Yang^a^, Yuanfeng Zhu^a^, Yongling Lu^a^, Qian Chen^a^, Hong Zhou ^b^, and Jiang Zheng^a^,*

*^a^Medical Research Center, Southwest Hospital, the Third Military Medical University, Chongqing, 400038, China*

*^b^Department of Pharmacology, College of Pharmacy, the Third Military Medical University, Chongqing 400038, China*

^*^Corresponding author: Jiang Zheng. Address: Medical Research Center, Southwest Hospital, the Third Military Medical University, Gaotanyan Street 30, Shapingba District, Chongqing 400038, P.R. China. Tel: +86 23 68765971, Fax: +86 23 68765468, E-mail: zhengj99219@163.com

**Table S1.** Real-time PCR primers

| **Name** | **Sequences** |
| --- | --- |
| β-actin | F: 5’-GGGAAATCGTGCGTGACATCAAAG-3’ |
|  | R: 5’-CATACCCAAGAAGGAAGGCTGGAA-3’ |
| TNF-α | F: 5’-CAGGTTCTGTCCCTTTCACTCACT-3’ |
|  | R: 5’-GTTCAGTAGACAGAAGAGCGTGGT-3’ |
| IL-6 | F: 5’- TGGAGTACCATAGCTACCTGGAGT -3’ |
|  | R: 5’- TCCT-TAGCCACTCCTTCTGTGACT-3’ |
| TLR4 | F: 5’- AAGGCATGGCATGGCTTACAC -3’ |
|  | R: 5’- GGCCAATTTTGTCTCCACAGC -3’ |
| iNOS | F: 5’- TCCTACACCACACCAAAC -3’ |
|  | R: 5’- CTCCAATCTCTGCCTATCC -3’ |
| COX2 | F: 5’- TAGCAGATGACTGCCCAACT -3’ |
|  | R: 5’- CACCTCTCCACCAATGACCT -3’ |
| NOX2 | F: 5’- TTCAACGTTGAGTGGTGTGT -3’ |
|  | R: 5’- ATGTACTGTCCCACCTCCAT -3’ |
| ATF3 | F: 5’- GAGCTGAGATTCGCCATCCA-3’ |
|  | R: 5’-CCGCCTCCTTTTCCTCTCAT-3’ |


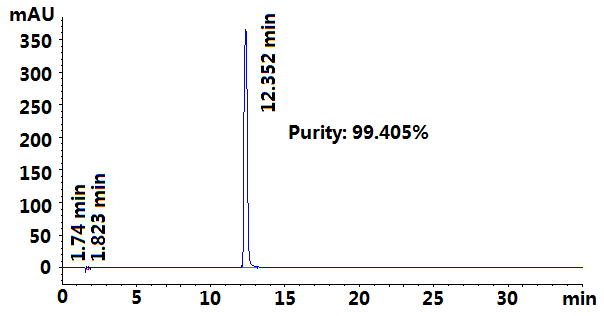


**Figure S1. Purity study of naringenin determined by HPLC.** Naringenin was dissolved in DMSO (1 mg/ml), sampled using an Agilent 1200 analytic HPLC system, and separated by gradient elution with 0.1% formic acid (A) and methanol (B) for 30 min (10% B - 100% B). The purity was determined by the peak area ratio analysis via the DAD detector.

**
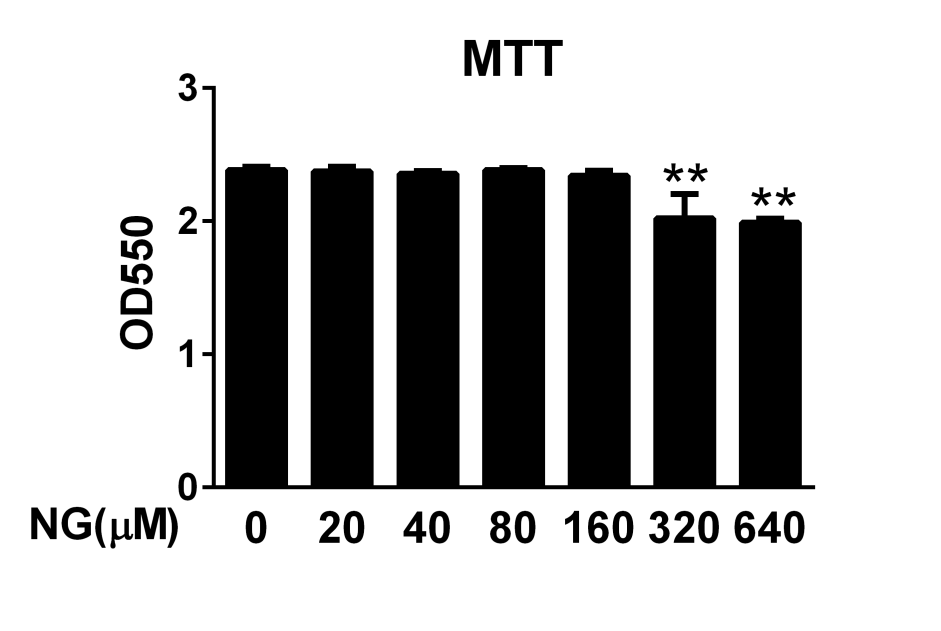
**

**Figure S2. Cytotoxicity detection of naringenin by the MTT assay.** RAW 264.7 cells were seeded in 96-well plates, and 0-640 μM naringenin was added (NG) for 24 h. The cell viability was detected by an MTT assay (n=6). **: *P*<0.01 vs NG (0 μM). Naringenin is abbreviated as NG.

**Figure S3. Naringenin inhibits TNF-α and IL-6 production in LPS-stimulated** **murine peritoneal macrophages and BMDMs in a dose-dependent manner.** Murine peritoneal macrophages (A) or BMDMs (B) were treated with 80 μM NG, 100 ng/ml LPS or LPS with NG (2.5, 5, 10, 20, 40 and 80 μM) for 12 h. Supernatant TNF-α and IL-6 levels were detected by ELISA (n=4). **: *P* <0.01 vs LPS (TNF-α), #: *P* <0.05; ##: P<0.01 vs LPS (IL-6). Naringenin is abbreviated as NG.

**Figure S4. Naringenin attenuates** **TNF-α and IL-6 mRNA expression in LPS-stimulated BMDMs.** Cells were treated with LPS alone or with NG (20, 40 and 80 μM) for 4 h. TNF-α and IL-6 mRNA was detected by real-time PCR. **: *P*<0.01 *vs* LPS (TNF-α), ##: *P*<0.01 *vs* LPS (IL-6). Naringenin is abbreviated as NG.

**
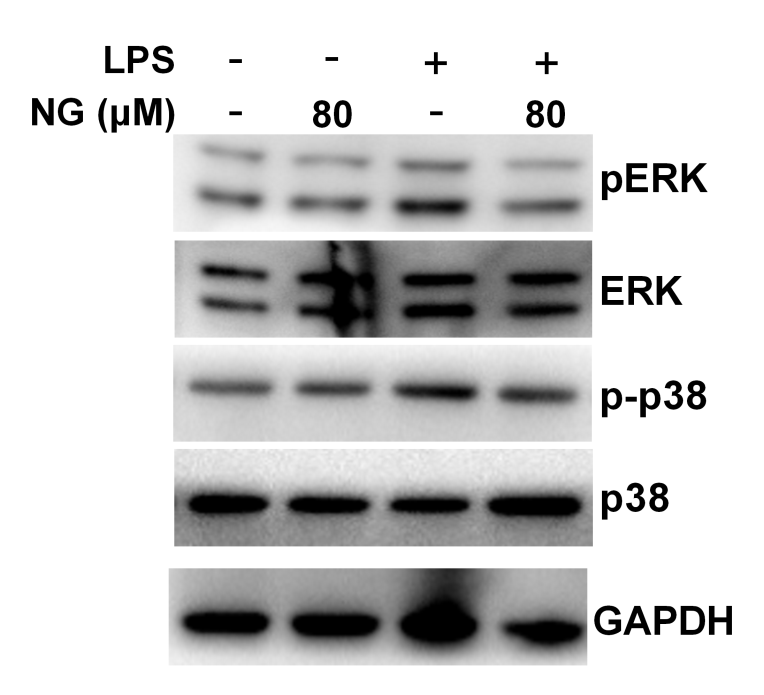
**

**Figure S5. Naringenin inhibits phosphorylation of ERK and p38 in LPS-stimulated BMDMs.** Cells were untreated or treated with 80 μM NG, LPS or LPS plus 80 μM NG. The protein levels of p-ERK, ERK, p-p38 and p38 were detected by Westernblot 0.5 h after treatment (B). Naringenin is abbreviated as NG.

**Figure S6. Naringenin upregulated ATF3 expression and AMPK phosphorylation in LPS-stimulated BMDMs.** Cells were untreated or treated with 80 μM NG, LPS or LPS plus 80 μM NG. The mRNA expression of ATF3 was detected 4h after treatment (A). The protein levels of p-AMPKα and AMPKα were detected by Westernblot 1 h after treatment (B). **:*P*<0.01. Naringenin is abbreviated as NG.

**Figure S7. Naringenin does not inhibit the production of IFN-β and RANTES in LPS challenged mice.** Mice were treated as in Figure 7. Serum levels of IFN-β and RANTES were detected 12 h after injection. Naringenin is abbreviated as NG.

**Figure S8 Uncropped versions of the blots in Figure 4, 5 and 7.**
